# Does sympathetic vasoconstriction contribute to metabolism: Perfusion matching in exercising skeletal muscle?

**DOI:** 10.3389/fphys.2022.980524

**Published:** 2022-09-12

**Authors:** Darren S. DeLorey, Philip S. Clifford

**Affiliations:** ^1^ Faculty of Kinesiology, Sport, and Recreation, University of Alberta, Edmonton, AB, Canada; ^2^ College of Applied Health Sciences, University of Illinois at Chicago, Chicago, IL, United States

**Keywords:** blood flow, exercise, sympatholysis, blood pressure, autonomic nervous system

## Abstract

The process of matching skeletal muscle blood flow to metabolism is complex and multi-factorial. In response to exercise, increases in cardiac output, perfusion pressure and local vasodilation facilitate an intensity-dependent increase in muscle blood flow. Concomitantly, sympathetic nerve activity directed to both exercising and non-active muscles increases as a function of exercise intensity. Several studies have reported the presence of tonic sympathetic vasoconstriction in the vasculature of exercising muscle at the onset of exercise that persists through prolonged exercise bouts, though it is blunted in an exercise-intensity dependent manner (functional sympatholysis). The collective evidence has resulted in the current dogma that vasoactive molecules released from skeletal muscle, the vascular endothelium, and possibly red blood cells produce local vasodilation, while sympathetic vasoconstriction restrains vasodilation to direct blood flow to the most metabolically active muscles/fibers. Vascular smooth muscle is assumed to integrate a host of vasoactive signals resulting in a precise matching of muscle blood flow to metabolism. Unfortunately, a critical review of the available literature reveals that published studies have largely focused on bulk blood flow and existing experimental approaches with limited ability to reveal the matching of perfusion with metabolism, particularly between and within muscles. This paper will review our current understanding of the regulation of sympathetic vasoconstriction in contracting skeletal muscle and highlight areas where further investigation is necessary.

## Introduction

Exercise presents a substantial challenge to the cardiovascular system. In order to sustain exercise, the CV system must satisfy the competing demands of adequately perfusing active skeletal muscle while simultaneously maintaining systemic blood pressure (Joyner and Casey, 2015; Mueller et al., 2017). Cardiac output and limb blood flow increase in proportion to exercise intensity suggesting that muscle blood flow and O_2_ delivery are matched to metabolic demand (Joyner and Casey, 2015; Mueller et al., 2017). Using radioactive microspheres, Armstrong and Laughlin (1984) demonstrated that blood flow was distributed unequally among muscles of the hindlimb during treadmill running. Moreover, the blood flow pattern was consistent with muscle recruitment patterns and metabolism, suggesting that blood flow was precisely matched to metabolic rate in individual muscles during incremental treadmill exercise.

**FIGURE 1:**
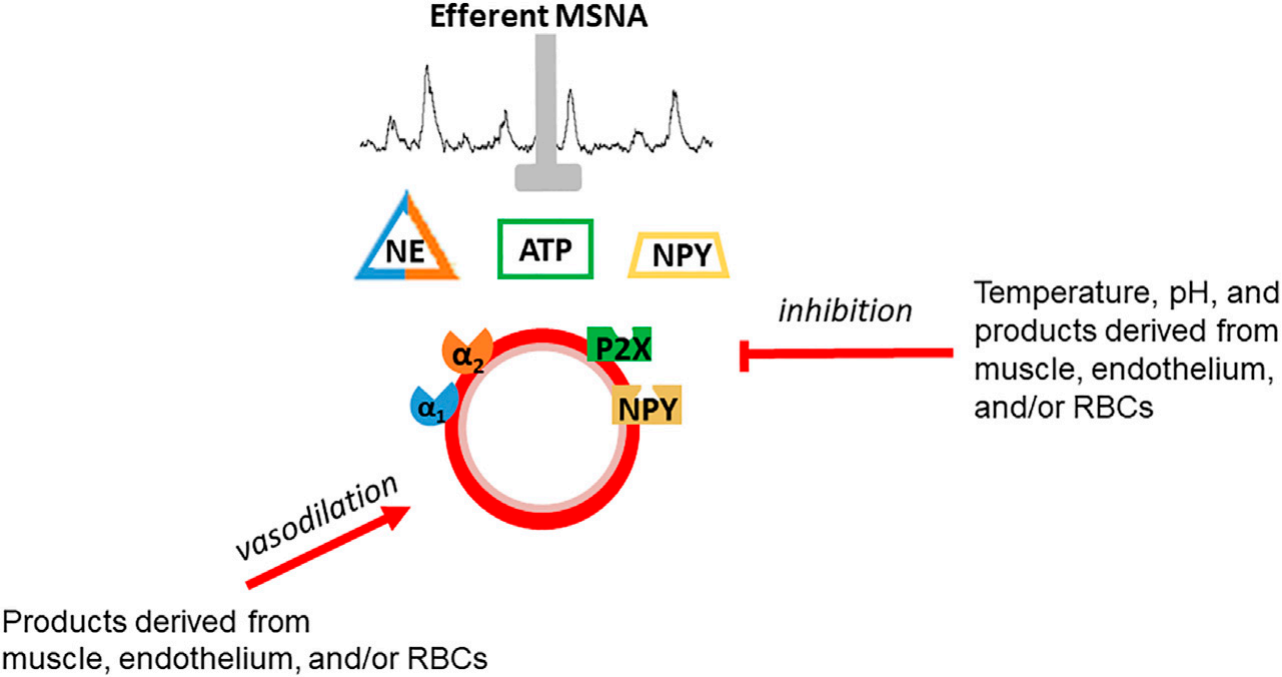
Schematic depiction of functional sympatholysis which is postulated to be due to inhibition of sympathetic vasoconstriction of by vasoactive molecules released from skeletal muscle, the vascular endothelium, or red blood cells. Temperature and pH also contribute. This phenomenon is distinct from vasodilation although some of the same substances are thought to be involved in vasodilation and sympatholysis.

Skeletal muscle vascular beds possess a profound ability to vasodilate in response to exercise (Andersen and Saltin, 1985). Indeed, vasodilation of a large proportion of muscle mass or maximal vasodilation of a smaller proportion of skeletal muscle (estimated by Andersen and Saltin to be 1/3 of muscle mass in sedentary individuals) may exceed the pumping capacity of the heart to perfuse active muscle and maintain systemic blood pressure (Andersen and Saltin, 1985). Vasoconstriction of inactive tissues helps redistribute blood flow to active skeletal muscle to avoid outstripping cardiac output. This vasoconstriction is a result of an exercise intensity-dependent increase in efferent sympathetic nerve system (SNS) activity (Savard et al., 1987; O’Hagan et al., 1993; DiCarlo et al., 1996; Boulton et al., 2018). There is evidence of tonic vasoconstriction in both inactive tissues and active skeletal muscle during dynamic exercise (Buckwalter et al., 1997; Buckwalter and Clifford, 2001). However, the effectiveness of SNS activity to produce vasoconstriction is blunted in active muscle and declines as a function of exercise-intensity (Buckwalter and Clifford, 2001; Thomas and Segal, 2004). This contraction-mediated inhibition of sympathetic vasoconstriction was termed functional sympatholysis by Remensnyder et al. (1962). While the precise mechanism(s) responsible for sympatholysis have not been fully elucidated, the available evidence suggests that vasoactive molecules released from skeletal muscle, the vascular endothelium, and possibly red blood cells decrease the responsiveness of post-synaptic sympathetic receptors (Figure 1) (Thomas and Segal, 2004; Just et al., 2016; Mueller et al., 2017; DeLorey, 2021). The current dogma, oft repeated over the past several decades, is that functional sympatholysis is responsible for directing blood flow to the most metabolically active muscles/fibers.

Unfortunately, there is limited experimental evidence to demonstrate that such an integrated control system is involved in blood flow to metabolism matching in active muscle. This paper will review our current understanding of the regulation of sympathetic vasoconstriction in resting and contracting skeletal muscle with a particular focus on the role of sympathetic vasoconstriction in perfusion to metabolism matching during exercise. We will also discuss the need for further investigation in this area and emerging technologies that may advance our understanding of metabolism:perfusion matching during exercise.

## Sympathetic restraint of skeletal muscle blood flow

Acute exercise evokes an exercise-intensity dependent increase in efferent sympathetic nervous system activity in animals (DiCarlo et al., 1996) and humans (Savard et al., 1987; Savard et al., 1989; Boulton et al., 2018). Although the increase in sympathetic outflow would be expected to elicit vasoconstriction, Donald et al. (1970) reported that hindlimb blood flow was not altered in dogs running on a treadmill following sympathectomy. In contrast, Peterson et al. (1988) reported that acute sympathectomy resulted in higher total hindlimb blood flow in rats during mild-intensity treadmill exercise. Moreover, sympathectomy increased blood flow to knee extensor muscles unequally, with flow increasing from 33 to 79% in individual muscles, indicating that sympathetic vasoconstriction is instrumental to the distribution of muscle blood flow during exercise (Peterson et al., 1988). Using intra-arterial injection of alpha(α)-receptor antagonists, O’Leary et al. (1997) and Buckwalter et al. (1997) reported that acute α_1_ adrenergic receptor blockade increased hindlimb vascular conductance in dogs at rest and during exercise, indicating the presence of tonic α_1_ adrenergic receptor mediated restraint of skeletal muscle blood flow. A later study reported that there is also tonic α_2_-adrenergic receptor mediated vasoconstriction in exercising canine skeletal muscles (Buckwalter and Clifford, 1999). Furthermore, the magnitude of both α_1_ and α_2_ adrenergic receptor mediated vasoconstriction declined as a function of exercise intensity (Buckwalter and Clifford, 1999). Buckwalter et al. (2004a); Buckwalter et al. (2004b) also reported that non-adrenergic receptors produce tonic vasoconstriction during exercise. Specifically, injection of purinergic and neuropeptide Y (NPY) Y1 receptor antagonists during exercise increased hindlimb vascular conductance in dogs running on a motorized treadmill, indicating the presence of tonic NPY and purinergic receptor mediated vasoconstriction in exercising skeletal muscle (Buckwalter et al., 2003; Buckwalter et al., 2004a). NPY and purinergic receptor antagonism produced a smaller increase in vascular conductance during exercise compared to rest, indicating that muscle contraction also blunted non-adrenergic receptor mediated vasoconstriction (Buckwalter et al., 2003; Buckwalter et al., 2004a). Subsequently, DeLorey et al. (2006) reported that tonic α-adrenergic receptor mediated vasoconstriction was maintained throughout prolonged exercise. The fact that tonic vasoconstriction declined as exercise intensity increased and that α_2_ receptor activity was reduced at low intensities, whereas α_1_ receptor activity was reduced only at a heavy intensity suggests that muscular contraction may blunt sympathetic vasoconstriction in a receptor dependent manner (Buckwalter and Clifford, 2001).

Receptor specific modulation of vasoconstriction during exercise may be related to the distribution of post-synaptic receptors within the vascular tree as receptors positioned on distal branches of the vascular tree would be in closer proximity to the interstitial environment exposing them to larger concentrations of vasoactive molecules that may oppose sympathetic vasoconstriction. Data from the rat cremaster muscle indicate that α_2_-adrenergic receptors are localized to small, distal secondary and tertiary arterioles, whereas α_1_-adrenergic receptors are primarily located on larger proximal, arterioles (Faber, 1988; McGillivray-Anderson and Faber, 1990; Ohyanagi et al., 1992; Tateishi and Faber, 1995). However, in the mouse gluteus maximus muscle the functional distribution of α_2_ receptors was greater in proximal (1A) arterioles, while the functional distribution of α_1_ receptors was greater in distal (3A) arterioles (Moore et al., 2010). A recent study by Al-Khazraji et al. (2015) in the rat gluteus maximus muscle suggests that α-adrenoreceptors primarily regulate resistance in proximal arterioles (branch order 1A–3A), whereas purinergic and peptidergic receptors constrict more distal arteries (branch orders 4A and 5A). Overall, these data suggest the possibility that adrenergic receptor mediated vasoconstriction may regulate flow between muscles and non-adrenergic receptor mediated vasoconstriction may be important to the distribution of flow within muscles. To our knowledge, no studies have investigated the distribution of post-synaptic receptors in humans. Because the evidence from rodent studies shows that the distribution of receptors varies between skeletal muscles, it is important not to make assumptions about receptor location in human skeletal muscle.

Heinonen et al. (2013) used positron emission tomography (PET) and a single-leg knee-extension exercise model to investigate the distribution of blood flow in the thigh at rest and during exercise. Non-selective pharmacological inhibition of postsynaptic alpha-adrenergic receptors increased conductance in inactive muscles, but had no effect on blood flow to active muscles during exercise, suggesting that tonic vasoconstriction in inactive muscle contributes to the distribution of limb blood flow from inactive to active muscle.

In a separate study, Heinonen et al. (2012) investigated how exercise influences blood flow to a resting limb. During very-light intensity single-leg knee-extension exercise, total blood flow to the contralateral inactive leg did not change from pre-exercise levels. However, at the highest exercise intensity employed, flow within the inactive leg was redistributed between the hamstring and quadriceps muscle groups and flow heterogeneity (calculated as the coefficient of variation in muscle blood flow between regions of muscle imaged) increased. Since muscle sympathetic nerve activity (MSNA) declines in response to mild-intensity single-leg exercise (knee-extension or cycling) (Saito and Mano, 1991; Ray, 1993; Saito et al., 1993; Saito et al., 1997; Ichinose et al., 2008; Katayama et al., 2011) a withdrawal of vasoconstrictor tone may have contributed to the observed redistribution of flow between muscles of the inactive leg.

In summary, the available evidence, while not conclusive, demonstrates the presence of tonic adrenergic and non-adrenergic receptor mediated vasoconstriction in the skeletal muscle vascular bed during exercise. Tonic vasoconstriction is maintained throughout prolonged exercise, however the magnitude of tonic vasoconstriction appears to decline as a function of exercise intensity in a receptor-specific manner. Finally, the limited experimental evidence available demonstrates that tonic vasoconstriction contributes to the distribution of limb blood flow during exercise, with data from rodent studies suggesting an important role in matching blood flow to metabolism, whereas the only study available data in humans reported no effect of non-selective α-adrenergic receptor blockade on blood flow to active muscle.

## Sympathetic vasoconstrictor responsiveness and sympatholysis

Although sympathetic restraint of active skeletal muscle blood flow has been established, it has also been demonstrated that the effectiveness of sympathetic outflow to produce vasoconstriction is attenuated in active compared to resting skeletal muscle (functional sympatholysis) (Remensnyder et al., 1962; Thomas et al., 1994; Ruble et al., 2000; Buckwalter et al., 2001; DeLorey et al., 2002; Rosenmeier et al., 2003). As mentioned previously, the mechanism of sympatholysis is thought to involve the release of a vasoactive molecule from the endothelium, skeletal muscle, or possibly red blood cells that blunts the responsiveness of postsynaptic sympathetic receptors in contracting muscle. Indeed, considerable evidence suggests that the responsiveness of post-synaptic adrenergic receptors declines during exercise (Remensnyder et al., 1962; McGillivray-Anderson and Faber, 1990; Thomas et al., 1994; Ruble et al., 2000; Buckwalter et al., 2001; Ruble et al., 2002; Tschakovsky et al., 2002; Rosenmeier et al., 2003; DeLorey et al., 2012). McGillivray-Anderson and Faber (1990) reported a decline in adrenergic receptor responsiveness during contraction of the rat cremaster muscle, and that α_2-_adrenergic receptor mediated vasoconstriction was more easily inhibited than α_1-_receptor mediated constriction. In the rat hindlimb, muscle contraction blunted vasoconstriction to lumbar sympathetic nerve stimulation in glycolytic, but not oxidative skeletal muscle (Thomas et al., 1994). Consistent with the findings of Anderson and Faber, the attenuated vasoconstrictor responsiveness in glycolytic muscle was mediated by reduced responsiveness of α_2_-adrenergic receptors (Thomas et al., 1994).

In a series of studies in chronically instrumented dogs, Buckwalter et al. (1998); Buckwalter et al. (2001); Buckwalter et al. (2003); Buckwalter et al. (2004a) demonstrated that α-adrenergic, peptidergic and purinergic receptor responsiveness to selective agonists declined during exercise. Buckwalter et al. (2001), demonstrated that the vascular response to intra-arterial infusion of the selective α_1_-adrenergic receptor agonist, phenylephrine, was only reduced during heavy-intensity exercise, whereas the vascular response to the selective α_2_-adrenegic receptor agonist, clonidine, was diminished during mild- and heavy-intensity exercise. P2X-receptor responsiveness was attenuated during heavy-intensity exercise, whereas NPY-Y1 receptor responsiveness was blunted across exercise intensities (Buckwalter et al., 2003; Buckwalter et al., 2004a). Ruble et al. (2002) reported that the vasoconstrictor response to tyramine evoked release of the neurotransmitter norepinephrine was attenuated in dogs running on treadmill in an exercise intensity dependent manner. Similarly, Tschakovsky et al. (2002) reported exercise-intensity dependent attenuation of tyramine evoked vasoconstriction during rhythmic handgrip exercise in humans. VanTeeffelen and Segal (2003) reported that vasoconstrictor responsiveness of feed arteries and 1st order arterioles to sympathetic activation was maintained during skeletal muscle contraction, whereas responsiveness of 2nd and 3rd order arterioles was blunted. The blunting of vasoconstriction in distal, but not proximal, vascular segments suggests that local modulation of vasoconstriction may serve to match perfusion to metabolism within contracting muscles.

In humans, Wray et al. (2004) reported that vasoconstrictor responsiveness to the selective α_1_ adrenergic receptor agonist phenylephrine was maintained during mild intensity knee-extension exercise, but became blunted at heavier exercise intensities, whereas α_2_ adrenergic receptor responsiveness declined throughout a range of exercise intensities. In contrast, Rosenmeier et al. (2003) reported that α_1_-adrenergic and α_2_-adrenergic receptor responsiveness declined by a similar magnitude during handgrip exercise.

Since NE may also bind to beta (β)-adrenergic receptors, it is theoretically possible that NE binding to β-adrenergic receptors may oppose SNS mediated vasoconstriction. However, sympathetic vasoconstrictor responsiveness was not augmented following β-adrenergic receptor blockade in male and female rats, indicating that β-adrenergic receptors do not oppose sympathetic vasoconstriction in contracting skeletal muscle or contribute to functional sympatholysis (Cooper et al., 2019; Cooper et al., 2021).

Collectively, the available data related to changes in receptor responsiveness during exercise suggest that the distribution of post-synaptic receptors between muscles and in different vascular segments combined with muscle recruitment patterns may result in local modulation of vascular resistance that would serve to distribute blood flow between and within muscles and facilitate perfusion to metabolism matching during exercise. However, the definitive study to demonstrate this has not been completed.

The cellular mechanism(s) responsible for a decline in receptor responsiveness during exercise has not been fully elucidated, however the vasoactive molecule nitric oxide (NO) and intravascular adenosine triphosphate (ATP) have been the focus of investigation (Ohyanagi et al., 1992; Thomas and Victor, 1998; Dinenno and Joyner, 2003; Buckwalter et al., 2004c; Rosenmeier et al., 2004; Kirby et al., 2008; Kirby et al., 2011; Jendzjowsky and DeLorey, 2013a; Jendzjowsky and DeLorey, 2013c; Jendzjowsky et al., 2014a; Hearon et al., 2017; Just and DeLorey, 2017). NO is produced by the reaction of L-arginine with O_2_ catalyzed by the enzyme NO synthase (NOS), which exists in endothelial (eNOS), neuronal (nNOS), and inducible (iNOS) isoforms (Kobzik et al., 1994). Tetrahydrobiopterin (BH4) is an essential cofactor required for NO production by NOS enzymes (Blackwell et al., 2004; Katusic et al., 2009; Forstermann and Sessa, 2012). eNOS and nNOS are constitutively expressed in several tissues, including skeletal muscle and the endothelium (Kobzik et al., 1994; Frandsen et al., 1996; Boulanger et al., 1998; Bachetti et al., 2004). Several studies have reported increased sympathetic vasoconstrictor responsiveness and impaired sympatholysis following non-selective pharmacological blockade of NO production, indicating that NO blunts sympathetic vasoconstriction in contracting muscle (Ohyanagi et al., 1992; Häbler et al., 1997; Thomas and Victor, 1998; Jendzjowsky and DeLorey, 2013c). In contrast to these findings, other studies in humans and canines have reported that NOS blockade did not affect the magnitude of sympatholysis (Dinenno and Joyner, 2003; Buckwalter et al., 2004c). Acute upregulation of NO bioavailability can be accomplished by infusing the essential cofactor BH4. This approach was employed by Jendzjowsky et al. (2014b) who reported reduced sympathetic vasoconstrictor responsiveness during contraction of the rat hindlimb following BH_4_ administration. A reduced ability to inhibit sympathetic vasoconstriction during muscular contraction has been reported in humans with duchenne muscular dystrophy (Sander et al., 2000) and in genetically modified mice (Thomas et al., 2003) that do not express nNOS. In healthy sedentary rats, NO derived from eNOS and nNOS contributed equally to the inhibition of sympathetic vasoconstriction in contracting muscle (Jendzjowsky and DeLorey, 2013a).

Exercise training has also been used to investigate sympatholysis (Jendzjowsky and DeLorey, 2013b; Jendzjowsky and DeLorey, 2013c; Jendzjowsky et al., 2014a; Mizuno et al., 2014; Mortensen et al., 2014; Just and DeLorey, 2016; Just et al., 2016; Cooper et al., 2021). In rats, exercise training enhanced sympatholysis through an NO dependent mechanism (Jendzjowsky and DeLorey, 2013c; Mizuno et al., 2014). Enhanced NO dependent sympatholysis following exercise training appears to be predominantly mediated by upregulation of nNOS because selective nNOS blockade abolished the enhanced sympatholysis in exercise trained rats and subsequent non-selective NOS blockade did not further reduce sympatholysis (Jendzjowsky et al., 2014a). nNOS enzyme expression was elevated in the hindlimb (predominantly in fast-twitch lateral gastrocnemius muscle) of exercise trained rats, suggesting a causal link between NOS enzyme expression and NO mediated vascular function (Jendzjowsky et al., 2014a). However, studies by Heinonen et al. (2011); Heinonen et al. (2017) indicate that non-selective NOS inhibition does not alter blood flow to active and inactive muscle during mild-intensity, single-leg knee-extension exercise. In contrast, several studies in rodents indicate that NO contributes to the distribution of muscle blood flow during exercise, and is particularly important to the distribution of flow during intense exercise (Hirai et al., 1994; Musch et al., 2001; Copp et al., 2010; Copp et al., 2013).

Given that NOS enzymes are expressed differentially across skeletal muscles (Kobzik et al., 1994; Jendzjowsky et al., 2014a), it seems plausible that local NOS expression and NO-dependent sympatholysis may contribute to perfusion to metabolism matching during exercise in an exercise intensity dependent manner.

Intravascular infusion of exogenous ATP has repeatedly been shown to inhibit sympathetic vasoconstriction (Rosenmeier et al., 2004; Kirby et al., 2008; Kirby et al., 2011; Hearon et al., 2017). Interestingly, combined blockade of the primary pathways through which ATP produces vasodilation (NO, prostaglandins and K^+^ channels) does not alter the ability of exogenous ATP to blunt α-adrenergic receptor mediated vasoconstriction (Hearon et al., 2017). While intravascular and interstitial levels of ATP both increase in response to exercise (Mortensen et al., 2009; Crecelius et al., 2013; Hearon et al., 2017), it is presently unknown whether endogenously produced ATP can blunt sympathetic vasoconstriction. Because a selective P2Y receptor antagonist does not currently exist, the definitive study in this area is not technically feasible.

## Existing evidence that sympathetic vasoconstriction redistributes blood flow

Thomas et al. (1994) reported that vasoconstriction evoked by direct stimulation of the lumbar sympathetic chain in anesthetized rats was inhibited to a greater extent in contracting muscles composed predominantly of glycolytic fibers compared with muscle composed of oxidative fibers. Consistent with the findings of Thomas et al., Horiuchi et al. (2014) reported that sympathoexcitation *via* a cold-pressor test produced a smaller decrease in muscle oxygenation the gastrocnemius compared with the soleus muscle during mild-intensity plantar flexion exercise, suggesting a greater blunting of sympathetic vasoconstriction in glycolytic, compared with oxidative muscle. Heinonen et al. (2013) used positron emission tomography (PET) to investigate the distribution of blood flow in the thigh at rest and during exercise. Non-selective pharmacological inhibition of postsynaptic α-adrenergic receptors increased conductance in inactive muscles, but had no effect on blood flow to active muscles during exercise resulting in less effective matching of blood flow to metabolism. Collectively, these studies suggest that sympathetic vasoconstriction contributes to the distribution of blood flow between muscles and between muscle and other tissues during exercise.

## Future directions

It is apparent from the evidence presented above that, with a few exceptions, all of the studies of functional sympatholysis have studied bulk blood flow through a conduit artery and are incapable of demonstrating whether the observed sympatholysis contributes to matching of perfusion to metabolism within or between muscles. In the paper by Heinonen et al. (2013) femoral artery infusion of norepinephrine at rest decreased blood flow in muscles, bone, and adipose tissue of the human thigh. During exercise the blood flow and vascular conductance responses to norepinephrine infusion were markedly attenuated in the active quadriceps femoris and in bone, with no statistically significant change in the inactive hamstrings. Inhibition of α-adrenergic vasoconstriction with phentolamine had no effect on blood flow in the active quadriceps femoris, but increased blood flow in the inactive hamstrings, bone, and adipose tissue. These findings suggest that sympathetic nerve activity restrains blood flow in the inactive tissues to help maintain blood flow in the active muscle. The conclusion is strengthened by the fact blood flow was measured simultaneously in active and inactive muscles.

Unfortunately, existing studies have made the claim that sympathetic nerve activity aids redistribution of blood flow from inactive to active muscles without any experimental evidence. New approaches are needed to demonstrate that phenomenon. Several new technologies that might be useful include NIRS with indocyanine green (NIRS ICG), contrast enhanced ultrasound (CEU), and diffuse correlational spectroscopy (DCS/NIRS).

NIRS-ICG has been applied to investigation of blood flow in skeletal muscle and the results compare favorably with dye dilution MRI methods during knee extension exercise (Boushel et al., 2000). The technique provides a discrete measurement of blood flow which could be applied during steady-state exercise. If used in combination with injection of tyramine or reflex activation (e.g., cold pressor test) of the sympathetic nervous system, NIRS-ICG may provide insight into the distribution of limb blood flow and the role of sympathetic vasoconstriction in perfusion to metabolism matching during exercise. Drawbacks include the need for arterial injection of the tracer and the relatively small and superficial volume of tissue that is interrogated by the NIRS technique.

Contrast enhanced ultrasound requires infusion of microbubbles which are smaller than red blood cells to allow them to pass through the skeletal muscle microcirculation. As described by Dunford et al. (2018) an ultrasound transducer is positioned above the skeletal muscle of interest and the microbubbles reflect acoustic ultrasound energy during their passage through the muscle. Movement artifact is a concern, so it has so far only been applied in resting studies.

Diffuse correlational spectroscopy (DCS) is an emerging noninvasive technology that has been applied during exercise by several laboratories (Hammer et al., 2018; Tucker et al., 2019; Ichinose et al., 2021). Combining DCS with widely used near infrared spectroscopy (NIRS) allows continuous measurement of blood flow and has been validated against several standards including microspheres (Zhou et al., 2009) and arterial spin labelled MRI (Yu et al., 2007).

An example of how these techniques might be employed would be to apply DCS/NIRS sensors bilaterally to one limb that remains resting and the other limb which performs contractions across a range of exercise intensities. Sympathetic nerve activity could be activated reflexly to both limbs by using lower body negative pressure (LBNP) or cold pressor stimuli. Greater vasoconstriction in inactive skeletal muscle compared to active skeletal muscle would support redistribution of blood flow.

The above example suggests an experimental approach to demonstrate redistribution of blood flow between muscles. Finding a method to study redistribution of blood flow within a muscle to more active fibers is more problematic. The fundamental problem is that microvascular units are not precisely aligned with muscle motor units. Constriction of a terminal arteriole reduces perfusion to all the capillaries supplied by that arteriole which means that perfusion cannot be exclusively directed to active muscle fibers or a single fiber type (Fuglevand and Segal, 1997).

Although not discussed in this focused review, there is ample evidence that aging, sex, obesity and various disease states modulate sympatholysis and impairments in sympatholysis have functional consequences for exercise performance. Further exploration of the effects of sympatholysis in these populations/conditions is warranted.

## Conclusion

Our current understanding is that the matching of skeletal muscle blood flow to metabolism involves a complex interplay between local vasodilation and sympathetic vasoconstriction that restrains local vasodilation and directs blood flow to active muscles, while also maintaining total peripheral resistance and blood pressure.

The presence of tonic vasoconstriction in inactive tissue and exercising limbs is well established, and some studies have demonstrated that tonic vasoconstriction contributes to the distribution of blood flow within the limb during exercise. Numerous studies in a variety of experimental models have also demonstrated that sympathetic vasoconstriction is blunted in contracting muscle (sympatholysis). While investigators have done outstanding work to characterize the phenomenon of sympatholysis and identify some potential mechanisms involved in the process, direct experimental evidence that sympatholysis is involved in the matching of perfusion to metabolism is lacking.

Despite the sound logic that sympatholysis would facilitate the distribution of blood flow between and within muscles during exercise, currently available technology and experimental approaches make the definitive study to demonstrate that sympatholysis contributes to the matching of perfusion to metabolism infeasible in the dynamically exercising animal or human. Until there is new evidence provided, researchers should refrain from speculating that functional sympatholysis can redirect blood flow to active fibers within a muscle.

## References

[B1] Al-Khazraji B. K. Saleem A. Goldman D. Jackson D. N. (2015). From one generation to the next: a comprehensive account of sympathetic receptor control in branching arteriolar trees. J. Physiol. 593, 3093–3108. 10.1113/JP270490 25952132 PMC4532529

[B2] Andersen P. Saltin B. (1985). Maximal perfusion of skeletal muscle in man. J. Physiol. 366, 233–249. 10.1113/jphysiol.1985.sp015794 4057091 PMC1193029

[B3] Armstrong R. B. Laughlin M. H. (1984). Exercise blood flow patterns within and among rat muscles after training. Am. J. Physiol. 246, H59–H68. 10.1152/ajpheart.1984.246.1.H59 6696089

[B4] Bachetti T. Comini L. Curello S. Bastianon D. Palmieri M. Bresciani G. (2004). Co-expression and modulation of neuronal and endothelial nitric oxide synthase in human endothelial cells. J. Mol. Cell. Cardiol. 37, 939–945. 10.1016/j.yjmcc.2004.07.006 15522271

[B5] Blackwell K. A. Sorenson J. P. Richardson D. M. Smith L. A. Suda O. Nath K. (2004). Mechanisms of aging-induced impairment of endothelium-dependent relaxation: role of tetrahydrobiopterin. Am. J. Physiol. Heart Circ. Physiol. 287, H2448–H2453. 10.1152/ajpheart.00248.2004 15319209

[B6] Boulanger C. M. Heymes C. Benessiano J. Geske R. S. Levy B. I. Vanhoutte P. M. (1998). Neuronal nitric oxide synthase is expressed in rat vascular smooth muscle cells: activation by angiotensin II in hypertension. Circ. Res. 83, 1271–1278. 10.1161/01.res.83.12.1271 9851944

[B7] Boulton D. Taylor C. E. Green S. Macefield V. G. (2018). The metaboreflex does not contribute to the increase in muscle sympathetic nerve activity to contracting muscle during static exercise in humans. J. Physiol. 596, 1091–1102. 10.1113/JP275526 29315576 PMC5851889

[B8] Boushel R. Langberg H. Olesen J. Nowak M. Simonsen L. Bulow J. (2000). Regional blood flow during exercise in humans measured by near-infrared spectroscopy and indocyanine green. J. Appl. Physiol. 89, 1868–1878. 10.1152/jappl.2000.89.5.1868 11053338

[B9] Buckwalter J. B. Clifford P. S. (1999). Alpha -Adrenergic vasoconstriction in active skeletal muscles during dynamic exercise. Am. J. Physiol. 277, H33–H39. 10.1152/ajpheart.1999.277.1.H33 10409179

[B10] Buckwalter J. B. Clifford P. S. (2001). The paradox of sympathetic vasoconstriction in exercising skeletal muscle. Exerc. Sport Sci. Rev. 29, 159–163. 10.1097/00003677-200110000-00005 11688788

[B11] Buckwalter J. B. Hamann J. J. Clifford P. S. (2003). Vasoconstriction in active skeletal muscles: a potential role for P2X purinergic receptors? J. Appl. Physiol. 95, 953–959. 10.1152/japplphysiol.00173.2003 12766177

[B12] Buckwalter J. B. Hamann J. J. Kluess H. A. Clifford P. S. (2004a). Vasoconstriction in exercising skeletal muscles: a potential role for neuropeptide Y? Am. J. Physiol. Heart Circ. Physiol. 287, H144–H149. 10.1152/ajpheart.00071.2004 15210450

[B13] Buckwalter J. B. Mueller P. J. Clifford P. S. (1998). Alpha 1-Adrenergic-receptor responsiveness in skeletal muscle during dynamic exercise. J. Appl. Physiol. 85, 2277–2283. 10.1152/jappl.1998.85.6.2277 9843553

[B14] Buckwalter J. B. Mueller P. J. Clifford P. S. (1997). Sympathetic vasoconstriction in active skeletal muscles during dynamic exercise. J. Appl. Physiol. 83, 1575–1580. 10.1152/jappl.1997.83.5.1575 9375322

[B15] Buckwalter J. B. Naik J. S. Valic Z. Clifford P. S. (2001). Exercise attenuates alpha-adrenergic-receptor responsiveness in skeletal muscle vasculature. J. Appl. Physiol. 90, 172–178. 10.1152/jappl.2001.90.1.172 11133908

[B16] Buckwalter J. B. Taylor J. C. Hamann J. J. Clifford P. S. (2004b). Do P2X purinergic receptors regulate skeletal muscle blood flow during exercise? Am. J. Physiol. Heart Circ. Physiol. 286, H633–H639. 10.1152/ajpheart.00572.2003 14551053

[B17] Buckwalter J. B. Taylor J. C. Hamann J. J. Clifford P. S. (2004c). Role of nitric oxide in exercise sympatholysis. J. Appl. Physiol. 97, 417–423. 10.1152/japplphysiol.01181.2003 15020577

[B18] Cooper I. R. Just T. P. DeLorey D. S. (2019). β-Adrenoreceptors do not oppose sympathetic vasoconstriction in resting and contracting skeletal muscle of male rats. Appl. physiology, Nutr. metabolism = Physiologie appliquee, Nutr. metabolisme 44, 1230–1236. 10.1139/apnm-2019-0130 30951638

[B19] Cooper I. R. Liu S. DeLorey D. S. (2021). Effects of sex and exercise training on β-adrenoreceptor-mediated opposition of evoked sympathetic vasoconstriction in resting and contracting muscle of rats. J. Appl. Physiol. 130, 114–123. 10.1152/japplphysiol.00726.2020 33090912

[B20] Copp S. W. Hirai D. M. Schwagerl P. J. Musch T. I. Poole D. C. (2010). Effects of neuronal nitric oxide synthase inhibition on resting and exercising hindlimb muscle blood flow in the rat. J. Physiol. 588, 1321–1331. 10.1113/jphysiol.2009.183723 20176629 PMC2872736

[B21] Copp S. W. Holdsworth C. T. Ferguson S. K. Hirai D. M. Poole D. C. Musch T. I. (2013). Muscle fibre-type dependence of neuronal nitric oxide synthase-mediated vascular control in the rat during high speed treadmill running. J. Physiol. 591, 2885–2896. 10.1113/jphysiol.2013.251082 23507879 PMC3690692

[B22] Crecelius A. R. Kirby B. S. Richards J. C. Dinenno F. A. (2013). Mechanical effects of muscle contraction increase intravascular ATP draining quiescent and active skeletal muscle in humans. J. Appl. Physiol. 114, 1085–1093. 10.1152/japplphysiol.01465.2012 23429876 PMC3633434

[B24] DeLorey D. S. Clifford P. S. Mittelstadt S. Anton M. M. Kluess H. A. Tune J. D. (2012). The effect of aging on adrenergic and nonadrenergic receptor expression and responsiveness in canine skeletal muscle. J. Appl. Physiol. 112, 841–848. 10.1152/japplphysiol.00945.2011 22194325 PMC3311660

[B25] DeLorey D. S. Hamann J. J. Kluess H. A. Clifford P. S. Buckwalter J. B. (2006). Alpha-adrenergic receptor mediated restraint of skeletal muscle blood flow during prolonged exercise. J. Appl. Physiol. 100, 1563–1568. 10.1152/japplphysiol.01035.2005 16410381

[B26] DeLorey D. S. (2021). Sympathetic vasoconstriction in skeletal muscle: modulatory effects of aging, exercise training, and sex. Appl. Physiology, Nutr. Metabolism 46, 1437–1447. 10.1139/apnm-2021-0399 34348066

[B27] DeLorey D. S. Wang S. S. Shoemaker J. K. (2002). Evidence for sympatholysis at the onset of forearm exercise. J. Appl. Physiol. 93, 555–560. 10.1152/japplphysiol.00245.2002 12133864

[B28] DiCarlo S. E. Chen C. Y. Collins H. L. (1996). Onset of exercise increases lumbar sympathetic nerve activity in rats. Med. Sci. Sports Exerc. 28, 677–684. 10.1097/00005768-199606000-00006 8784755

[B29] Dinenno F. A. Joyner M. J. (2003). Blunted sympathetic vasoconstriction in contracting skeletal muscle of healthy humans: is nitric oxide obligatory? J. Physiol. 553, 281–292. 10.1113/jphysiol.2003.049940 12949223 PMC2343482

[B30] Donald D. E. Rowlands D. J. Ferguson D. A. (1970). Similarity of blood flow in the normal and the sympathectomized dog hind limb during graded exercise. Circ. Res. 26, 185–199. 10.1161/01.RES.26.2.185 5412534

[B31] Dunford E. C. Au J. S. Devries M. C. Phillips S. M. MacDonald M. J. (2018). Cardiovascular aging and the microcirculation of skeletal muscle: using contrast-enhanced ultrasound. Am. J. Physiol. Heart Circ. Physiol. 315, H1194–H1199. 10.1152/ajpheart.00737.2017 30074839 PMC6297816

[B32] Faber J. E. (1988). Effect of local tissue cooling on microvascular smooth muscle and postjunctional alpha 2-adrenoceptors. Am. J. Physiol. 255, H121–H130. 10.1152/ajpheart.1988.255.1.H121 2839993

[B33] Forstermann U. Sessa W. C. (2012). Nitric oxide synthases: regulation and function. Eur. Heart J. 33, 829–837. 10.1093/eurheartj/ehr304 21890489 PMC3345541

[B34] Frandsen U. Lopez-Figueroa M. Hellsten Y. (1996). Localization of nitric oxide synthase in human skeletal muscle. Biochem. Biophys. Res. Commun. 227, 88–93. 10.1006/bbrc.1996.1472 8858108

[B35] Fuglevand A. J. Segal S. S. (1997). Simulation of motor unit recruitment and microvascular unit perfusion: spatial considerations. J. Appl. Physiol. 83, 1223–1234. 10.1152/jappl.1997.83.4.1223 9338432

[B36] Hammer S. M. Alexander A. M. Didier K. D. Smith J. R. Caldwell J. T. Sutterfield S. L. (2018). The noninvasive simultaneous measurement of tissue oxygenation and microvascular hemodynamics during incremental handgrip exercise. J. Appl. Physiol. 124, 604–614. 10.1152/japplphysiol.00815.2017 29357515

[B37] Häbler H.-J. Wasner G. Jänig W. (1997). Attenuation of neurogenic vasoconstriction by nitric oxide in hindlimb microvascular beds of the rat *in vivo* . Hypertension 30, 957–961. 10.1161/01.HYP.30.4.957 9336400

[B38] Hearon C. M. Jr Richards J. C. Racine M. L. Luckasen G. J. Larson D. G. Joyner M. J. (2017). Sympatholytic effect of intravascular ATP is independent of nitric oxide, prostaglandins, Na(+)/K(+) -ATPase and KIR channels in humans. J. Physiol. 595, 5175–5190. 10.1113/JP274532 28590059 PMC5538228

[B39] Heinonen I. Duncker D. J. Knuuti J. Kalliokoski K. K. (2012). The effect of acute exercise with increasing workloads on inactive muscle blood flow and its heterogeneity in humans. Eur. J. Appl. Physiol. 112, 3503–3509. 10.1007/s00421-012-2329-5 22302377

[B40] Heinonen I. Saltin B. Hellsten Y. Kalliokoski K. K. (2017). The effect of nitric oxide synthase inhibition with and without inhibition of prostaglandins on blood flow in different human skeletal muscles. Eur. J. Appl. Physiol. 117, 1175–1180. 10.1007/s00421-017-3604-2 28432421

[B41] Heinonen I. Saltin B. Kemppainen J. Sipilä H. T. Oikonen V. Nuutila P. (2011). Skeletal muscle blood flow and oxygen uptake at rest and during exercise in humans: a pet study with nitric oxide and cyclooxygenase inhibition. Am. J. Physiol. Heart Circ. Physiol. 300, H1510–H1517. 10.1152/ajpheart.00996.2010 21257921

[B42] Heinonen I. Wendelin-Saarenhovi M. Kaskinoro K. Knuuti J. Scheinin M. Kalliokoski K. K. (2013). Inhibition of α-adrenergic tone disturbs the distribution of blood flow in the exercising human limb. Am. J. Physiol. Heart Circ. Physiol. 305, H163–H172. 10.1152/ajpheart.00925.2012 23666670

[B43] Hirai T. Visneski M. D. Kearns K. J. Zelis R. Musch T. I. (1994). Effects of NO synthase inhibition on the muscular blood flow response to treadmill exercise in rats. J. Appl. Physiol. 77, 1288–1293. 10.1152/jappl.1994.77.3.1288 7530705

[B44] Horiuchi M. Fadel P. J. Ogoh S. (2014). Differential effect of sympathetic activation on tissue oxygenation in gastrocnemius and soleus muscles during exercise in humans. Exp. Physiol. 99, 348–358. 10.1113/expphysiol.2013.075846 24163424

[B45] Ichinose M. Nakabayashi M. Ono Y. (2021). Rapid vasodilation within contracted skeletal muscle in humans: new insight from concurrent use of diffuse correlation spectroscopy and Doppler ultrasound. Am. J. Physiol. Heart Circ. Physiol. 320, H654–H667. 10.1152/ajpheart.00761.2020 33337963

[B46] Ichinose M. Saito M. Fujii N. Ogawa T. Hayashi K. Kondo N. (2008). Modulation of the control of muscle sympathetic nerve activity during incremental leg cycling. J. Physiol. 586, 2753–2766. 10.1113/jphysiol.2007.150060 18403425 PMC2536590

[B47] Jendzjowsky N. G. DeLorey D. S. (2013a). Role of neuronal nitric oxide in the inhibition of sympathetic vasoconstriction in resting and contracting skeletal muscle of healthy rats. J. Appl. Physiol. 115, 97–106. 10.1152/japplphysiol.00250.2013 23640592

[B48] Jendzjowsky N. G. DeLorey D. S. (2013b). Short-term exercise training augments 2-adrenoreceptor-mediated sympathetic vasoconstriction in resting and contracting skeletal muscle. J. Physiol. 591, 5221–5233. 10.1113/jphysiol.2013.257626 23940382 PMC3810820

[B49] Jendzjowsky N. G. DeLorey D. S. (2013c). Short-term exercise training enhances functional sympatholysis through a nitric oxide-dependent mechanism. J. Physiol. 591, 1535–1549. 10.1113/jphysiol.2012.238998 23297301 PMC3607171

[B50] Jendzjowsky N. G. Just T. P. DeLorey D. S. (2014a). Exercise training augments neuronal nitric oxide synthase-mediated inhibition of sympathetic vasoconstriction in contracting skeletal muscle of rats. J. Physiol. 592, 4789–4802. 10.1113/jphysiol.2014.278846 25194041 PMC4253477

[B51] Jendzjowsky N. G. Just T. P. Jones K. E. DeLorey D. S. (2014b). Acute tetrahydrobiopterin supplementation attenuates sympathetic vasoconstrictor responsiveness in resting and contracting skeletal muscle of healthy rats. Physiol. Rep. 2, e12164. Print 2014 Oct 1. 10.14814/phy2.12164 25318748 PMC4254091

[B52] Joyner M. J. Casey D. P. (2015). Regulation of increased blood flow (hyperemia) to muscles during exercise: a hierarchy of competing physiological needs. Physiol. Rev. 95, 549–601. 10.1152/physrev.00035.2013 25834232 PMC4551211

[B53] Just T. P. Cooper I. R. DeLorey D. S. (2016). Sympathetic vasoconstriction in skeletal muscle: Adaptations to exercise training. Exerc. Sport Sci. Rev. 44, 137–143. 10.1249/JES.0000000000000085 27433976

[B54] Just T. P. DeLorey D. S. (2016). Exercise training and α1-adrenoreceptor-mediated sympathetic vasoconstriction in resting and contracting skeletal muscle. Physiol. Rep. 4, e12707. 10.14814/phy2.12707 26869686 PMC4758927

[B55] Just T. P. DeLorey D. S. (2017). Sex differences in sympathetic vasoconstrictor responsiveness and sympatholysis. J. Appl. Physiol. 123, 128–135. 10.1152/japplphysiol.00139.2017 28473610 PMC5538817

[B56] Katayama K. Ishida K. Iwamoto E. Iemitsu M. Koike T. Saito M. (2011). Hypoxia augments muscle sympathetic neural response to leg cycling. Am. J. Physiol. Regul. Integr. Comp. Physiol. 301, R456–R464. 10.1152/ajpregu.00119.2011 21593431

[B57] Katusic Z. S. d’Uscio L. v. Nath K. A. (2009). Vascular protection by tetrahydrobiopterin: progress and therapeutic prospects. Trends Pharmacol. Sci. 30, 48–54. 10.1016/j.tips.2008.10.003 19042039 PMC2637534

[B58] Kirby B. S. Crecelius A. R. Voyles W. F. Dinenno F. A. (2011). Modulation of postjunctional alpha-adrenergic vasoconstriction during exercise and exogenous ATP infusions in ageing humans. J. Physiol. 589, 2641–2653. 10.1113/jphysiol.2010.204081 21486772 PMC3115831

[B59] Kirby B. S. Voyles W. F. Carlson R. E. Dinenno F. A. (2008). Graded sympatholytic effect of exogenous ATP on postjunctional alpha-adrenergic vasoconstriction in the human forearm: Implications for vascular control in contracting muscle. J. Physiol. 586, 4305–4316. 10.1113/jphysiol.2008.154252 18617568 PMC2652174

[B60] Kobzik L. Reid M. B. Bredt D. S. Stamler J. S. (1994). Nitric oxide in skeletal muscle. Nature 372, 546–548. 10.1038/372546a0 7527495

[B61] McGillivray-Anderson K. M. Faber J. E. (1990). Effect of acidosis on contraction of microvascular smooth muscle by alpha 1- and alpha 2-adrenoceptors. Implications for neural and metabolic regulation. Circ. Res. 66, 1643–1657. 10.1161/01.res.66.6.1643 1971536

[B62] Mizuno M. Iwamoto G. A. Vongpatanasin W. Mitchell J. H. Smith S. A. (2014). Exercise training improves functional sympatholysis in spontaneously hypertensive rats through a nitric oxide-dependent mechanism. Am. J. Physiol. Heart Circ. Physiol. 307, H242–H251. 10.1152/ajpheart.00103.2014 24816260 PMC4101645

[B63] Moore A. W. Jackson W. F. Segal S. S. (2010). Regional heterogeneity of α-adrenoreceptor subtypes in arteriolar networks of mouse skeletal muscle. J. Physiol. 588, 4261–4274. 10.1113/jphysiol.2010.194993 20807785 PMC3002455

[B64] Mortensen S. P. González-Alonso J. Nielsen J.-J. Saltin B. Hellsten Y. (2009). Muscle interstitial ATP and norepinephrine concentrations in the human leg during exercise and ATP infusion. J. Appl. Physiol. 107, 1757–1762. 10.1152/japplphysiol.00638.2009 19797688

[B65] Mortensen S. P. Nyberg M. Gliemann L. Thaning P. Saltin B. Hellsten Y. (2014). Exercise training modulates functional sympatholysis and α-adrenergic vasoconstrictor responsiveness in hypertensive and normotensive individuals. J. Physiol. 592, 3063–3073. 10.1113/jphysiol.2014.273722 24860173 PMC4214660

[B66] Mueller P. J. Clifford P. S. Crandall C. G. Smith S. A. Fadel P. J. (2017). “Integration of central and peripheral regulation of the circulation during exercise: acute and chronic adaptations,” in Comprehensive Physiology (New Jersey: Wiley), 103–151. 10.1002/cphy.c160040 29357126

[B67] Musch T. I. McAllister R. M. Symons J. D. Stebbins C. L. Hirai T. Hageman K. S. (2001). Effects of nitric oxide synthase inhibition on vascular conductance during high speed treadmill exercise in rats. Exp. Physiol. 86, 749–757. 10.1111/j.1469-445X.2001.tb00040.x 11698969

[B68] O’Hagan K. P. Bell L. B. Mittelstadt S. W. Clifford P. S. (1993). Effect of dynamic exercise on renal sympathetic nerve activity in conscious rabbits. J. Appl. Physiol. 74, 2099–2104. 10.1152/jappl.1993.74.5.2099 8335535

[B69] Ohyanagi M. Nishigaki K. Faber J. E. (1992). Interaction between microvascular alpha 1- and alpha 2-adrenoceptors and endothelium-derived relaxing factor. Circ. Res. 71, 188–200. 10.1161/01.res.71.1.188 1318795

[B70] O’Leary D. S. Robinson E. D. Butler J. L. (1997). Is active skeletal muscle functionally vasoconstricted during dynamic exercise in conscious dogs? AJP - Regul. Integr. Comp. Physiology 272, R386. Available at:http://ajpregu.physiology.org/cgi/content/abstract/272/1/R386 . 10.1152/ajpregu.1997.272.1.R386 9039033

[B71] Peterson D. F. Armstrong R. B. Laughlin M. H. (1988). Sympathetic neural influences on muscle blood flow in rats during submaximal exercise. J. Appl. Physiol. 65, 434–440. 10.1152/jappl.1988.65.1.434 3403486

[B72] Ray C. A. (1993). Muscle sympathetic nerve responses to prolonged one-legged exercise. J. Appl. Physiol. 74, 1719–1722. 10.1152/jappl.1993.74.4.1719 8514687

[B73] Remensnyder J. P. Mitchell J. H. Sarnoff S. J. (1962). Functional sympatholysis during muscular activity. Observations on influence of carotid sinus on oxygen uptake. Circ. Res. 11, 370–380. 10.1161/01.res.11.3.370 13981593

[B74] Rosenmeier J. B. Dinenno F. A. Fritzlar S. J. Joyner M. J. (2003). alpha1- and alpha2-adrenergic vasoconstriction is blunted in contracting human muscle. J. Physiol. 547, 971–976. 10.1113/jphysiol.2002.037937 12588902 PMC2342729

[B75] Rosenmeier J. B. Hansen J. Gonzalez-Alonso J. (2004). Circulating ATP-induced vasodilatation overrides sympathetic vasoconstrictor activity in human skeletal muscle. J. Physiol. 558, 351–365. 10.1113/jphysiol.2004.063107 15155791 PMC1664919

[B76] Ruble S. B. Valic Z. Buckwalter J. B. Clifford P. S. (2000). Dynamic exercise attenuates sympathetic responsiveness of canine vascular smooth muscle. J. Appl. Physiol. 89, 2294–2299. 10.1152/jappl.2000.89.6.2294 11090581

[B77] Ruble S. B. Valic Z. Buckwalter J. B. Tschakovsky M. E. Clifford P. S. (2002). Attenuated vascular responsiveness to noradrenaline release during dynamic exercise in dogs. J. Physiol. 541, 637–644. 10.1113/jphysiol.2001.014738 12042367 PMC2290325

[B78] Saito M. Mano T. (1991). Exercise mode affects muscle sympathetic nerve responsiveness. Jpn. J. Physiol. 41, 143–151. 10.2170/jjphysiol.41.143 1857017

[B79] Saito M. Sone R. Ikeda M. Mano T. (1997). Sympathetic outflow to the skeletal muscle in humans increases during prolonged light exercise. J. Appl. Physiol. 82, 1237–1243. 10.1152/jappl.1997.82.4.1237 9104861

[B80] Saito M. Tsukanaka A. Yanagihara D. Mano T. (1993). Muscle sympathetic nerve responses to graded leg cycling. J. Appl. Physiol. 75, 663–667. 10.1152/jappl.1993.75.2.663 8226466

[B81] Sander M. Chavoshan B. Harris S. A. Iannaccone S. T. Stull J. T. Thomas G. D. (2000). Functional muscle ischemia in neuronal nitric oxide synthase-deficient skeletal muscle of children with Duchenne muscular dystrophy. Proc. Natl. Acad. Sci. U. S. A. 97, 13818–13823. 10.1073/pnas.250379497 11087833 PMC17659

[B82] Savard G. K. Richter E. A. Strange S. Kiens B. Christensen N. J. Saltin B. (1989). Norepinephrine spillover from skeletal muscle during exercise in humans: role of muscle mass. Am. J. Physiol. 257, H1812–H1818. 10.1152/ajpheart.1989.257.6.H1812 2603969

[B83] Savard G. Strange S. Kiens B. Richter E. A. Christensen N. J. Saltin B. (1987). Noradrenaline spillover during exercise in active versus resting skeletal muscle in man. Acta Physiol. Scand. 131, 507–515. 10.1111/j.1748-1716.1987.tb08270.x 3442240

[B84] Tateishi J. Faber J. E. (1995). Inhibition of arteriole alpha 2- but not alpha 1-adrenoceptor constriction by acidosis and hypoxia *in vitro* . Am. J. Physiol. 268, H2068–H2076. 10.1152/ajpheart.1995.268.5.H2068 7771557

[B85] Thomas G. D. Hansen J. Victor R. G. (1994). Inhibition of alpha 2-adrenergic vasoconstriction during contraction of glycolytic, not oxidative, rat hindlimb muscle. AJP - Heart Circulatory Physiology 266, H920. Available at: http://ajpheart.physiology.org/cgi/content/abstract/266/3/H920 . 10.1152/ajpheart.1994.266.3.H920 7909201

[B86] Thomas G. D. Segal S. S. (2004). Neural control of muscle blood flow during exercise. J. Appl. Physiol. 97, 731–738. 10.1152/japplphysiol.00076.2004 15247201

[B87] Thomas G. D. Shaul P. W. Yuhanna I. S. Froehner S. C. Adams M. E. (2003). Vasomodulation by skeletal muscle-derived nitric oxide requires alpha-syntrophin-mediated sarcolemmal localization of neuronal Nitric oxide synthase. Circ. Res. 92, 554–560. 10.1161/01.RES.0000061570.83105.52 12600881

[B88] Thomas G. D. Victor R. G. (1998). Nitric oxide mediates contraction-induced attenuation of sympathetic vasoconstriction in rat skeletal muscle. J. Physiol. 506, 817–826. 10.1111/j.1469-7793.1998.817bv.x 9503340 PMC2230749

[B89] Tschakovsky M. E. Sujirattanawimol K. Ruble S. B. Valic Z. Joyner M. J. (2002). Is sympathetic neural vasoconstriction blunted in the vascular bed of exercising human muscle? J. Physiol. 541, 623–635. 10.1113/jphysiol.2001.014431 12042366 PMC2290331

[B90] Tucker W. J. Rosenberry R. Trojacek D. Chamseddine H. H. Arena-Marshall C. A. Zhu Y. (2019). Studies into the determinants of skeletal muscle oxygen consumption: novel insight from near-infrared diffuse correlation spectroscopy. J. Physiol. 597, 2887–2901. 10.1113/JP277580 30982990 PMC8024923

[B91] VanTeeffelen J. W. G. E. Segal S. S. (2003). Interaction between sympathetic nerve activation and muscle fibre contraction in resistance vessels of hamster retractor muscle. J. Physiol. 550, 563–574. 10.1113/jphysiol.2003.038984 12754308 PMC2343056

[B92] Wray D. W. Fadel P. J. Smith M. L. Raven P. Sander M. (2004). Inhibition of alpha-adrenergic vasoconstriction in exercising human thigh muscles. J. Physiol. 555, 545–563. 10.1113/jphysiol.2003.054650 14694145 PMC1664852

[B93] Yu G. Floyd T. F. Durduran T. Zhou C. Wang J. Detre J. A. (2007). Validation of diffuse correlation spectroscopy for muscle blood flow with concurrent arterial spin labeled perfusion MRI. Opt. Express 15, 1064–1075. 10.1364/OE.15.001064 19532334

[B94] Zhou C. Eucker S. A. Durduran T. Yu G. Ralston J. Friess S. H. (2009). Diffuse optical monitoring of hemodynamic changes in piglet brain with closed head injury. J. Biomed. Opt. 14, 034015. 10.1117/1.3146814 19566308 PMC3169814

